# Home-Based Sleep Sensor Measurements in an Older Australian Population: Before and during a Pandemic

**DOI:** 10.3390/s21185993

**Published:** 2021-09-07

**Authors:** Mahnoosh Kholghi, Claire M. Ellender, Qing Zhang, Yang Gao, Liesel Higgins, Mohanraj Karunanithi

**Affiliations:** 1 Health & Biosecurity, CSIRO, Brisbane, QLD 4029, Australia; qing.zhang@csiro.au (Q.Z.); yang.gao@csiro.au (Y.G.); Liesel.Higgins@csiro.au (L.H.); mohan.karunanithi@csiro.au (M.K.); 2 Department of Respiratory & Sleep Medicine, Princess Alexandra Hospital, 199 Ipswich Rd, Woolloongabba, QLD 4102, Australia; Claire.Ellender@health.qld.gov.au; 3 School of Medicine, University of Queensland, Brisbane, QLD 4006, Australia

**Keywords:** mattress-based device, longitudinal sleep monitoring, older adults, pandemic-related health

## Abstract

Older adults are susceptible to poor night-time sleep, characterized by short sleep duration and high sleep disruptions (i.e., more frequent and longer awakenings). This study aimed to longitudinally and objectively assess the changes in sleep patterns of older Australians during the 2020 pandemic lockdown. A non-invasive mattress-based device, known as the EMFIT QS, was used to continuously monitor sleep in 31 older adults with an average age of 84 years old before (November 2019–February 2020) and during (March–May 2020) the COVID-19, a disease caused by a form of coronavirus, lockdown. Total sleep time, sleep onset latency, wake after sleep onset, sleep efficiency, time to bed, and time out of bed were measured across these two periods. Overall, there was no significant change in total sleep time; however, women had a significant increase in total sleep time (36 min), with a more than 30-min earlier bedtime. There was also no increase in wake after sleep onset and sleep onset latency. Sleep efficiency remained stable across the pandemic time course between 84–85%. While this sample size is small, these data provide reassurance that objective sleep measurement did not deteriorate through the pandemic in older community-dwelling Australians.

## 1. Introduction

The events of 2020 and COVID-19, a disease caused by a form of coronavirus, have exposed all populations to unprecedented stressors, with older populations identified early in the pandemic as being at increased risk of adverse outcomes if infected with COVID-19 [1]. Older populations are known to have significantly increased risk of experiencing post-traumatic stress disorder (PTSD) and adjustment disorder symptoms as a result of natural disaster compared to younger individuals [2]. In a large cohort of 52,730 adult respondents in China, in early 2020, people over 60 years were found to have higher stress scores compared to middle-aged respondents [3]. Over 65-year-olds in many countries have experienced a deterioration in self-reported sleep quality and mental health since the announcement of the COVID-19 pandemic by the World Health Organization [4,5,6,7,8].

To date, most reports on sleep quality in older populations have been self-reported changes, using validated questionnaires such as the Pittsburgh Sleep Quality Index (PSQI) or Insomnia Severity Index (ISI). During the first Italian lockdown, a cohort of 2291 participants self-reported a significant increase in sleep latency on PSQI compared with previous normative data [7]. A different cohort of 1310 Italians reported going to bed, on average, 41 min later, with 52.4% of respondents reporting poor quality sleep, despite a 26-min increase in time spent in bed [8].

Self-reported sleep quality, however, can poorly correlate with measured total sleep time [9]. Longitudinal measurement of sleep is of interest in order to investigate whether perceptions and objective sleep measurements align. Awareness of sleep–wake cycles is particularly important during a crisis; for example to ensure care providers allocate staff for at the correct time for nightly care routines.

Consumer-grade sleep measurement devices can be categorized into three main classes: (i) wrist-based devices (e.g., Apple watch^®^ (Apple Inc., Los Altos, CA, USA), FitBit^®^ (Fitbit, Inc., San Francisco, CA, USA), Garmin^®^(Garmin Ltd., Schaffhausen, Switzerland), Amazfit^®^ (Zepp Health Corporation, Hefei, China), Polar^®^ (Polar Electro Oy, Kempele, Finland)), (ii) bedside devices (e.g., S+^®^ (ResMed, San Diego, CA, USA), Touch-Free Life Care^®^ (Bam Labs, Inc., Los Gatos, CA, USA)), and (iii) mattress-based devices (e.g., Beddit^®^ (Beddit Oy, Espoo, Finland), EarlySense Mattress^®^ (EarlySense, Ramat Gan, Israel), EMFIT QS (Emfit Ltd., Vaajakoski, Finland), Withings Sleep (Whithings, Issy-les-Moulineaux, France), Beautyrest^®^ (The Simmons Bedding Company, Atlanta, GA, USA)). With regards to validation, wrist-worn devices have been shown to estimate sleep onset latency within 10 min (level of agreement between −15.1 min to +23.2 min) [10] and have a >90% sensitivity in detecting sleep, with reasonable agreement in detecting rapid eye movement (REM) sleep (60–75%) [11]. Validation of these devices has been undertaken in adolescent cohorts [12], healthy adults, and in sleep-disordered patients [13]; however, little validation has been undertaken in the 65 years and older population. The discomfort of wearing a device at night for longitudinal data measurement can limit device acceptability. Mattress-based devices have the advantage of a “set-and-forget” approach, given that they are not intrusive, which helps enable access to longitudinal data.

One such method for longitudinal sleep monitoring is a mattress-based device known as the EMFIT QS (Quantified Sleep), developed and manufactured by Emfit, Finland. This ballistocardiograph (BCG) measures mechanical chest wall movements from heartbeat and respiration, from which sleep stage and time are inferred. The EMFIT QS heart rate (HR) and respiration rate (RR) measurements have been validated against the gold standard in-laboratory polysomnography (PSG) electrocardiogram and respiratory inductive plethysmography. Comparing EMFIT QS against PSG data collected from 33 patients showed that the 95% limits of agreement for HR were between −4.4 and 4.4 beats per minute (bpm), whereas for the RR, these limits were between −2.5 and 2.2 respirations per minute (rpm) [14], indicating highly accurate results. This device has not been assessed in a >65-year-old population.

Given the fears of older populations worldwide, we were interested in characterizing potential changes in sleep duration and circadian rhythm following the announcement of the 2020 pandemic. Thus, this study aimed to measure the difference in nighttime sleep before and during COVID-19 restrictions, for a cohort of community-dwelling adults over 65 years old. The hypothesis was that there would be a >10% reduction in total sleep time, increased wake after sleep onset, and reduced sleep efficiency comparing November 2019–February 2020 with March–May 2020.

## 2. Materials and Methods

### 2.1. Study Design

This study is a prospective cohort study, with data drawn as a sub-study of a larger parallel group stratified randomized controlled trial (RCT), called the Dementia and Aged Care Services project (DACS) trial [15]. DACS was conducted from November 2019 to November 2020 in Queensland, Australia, to assess the long-term benefits of utilizing the Smarter Safer Homes (SSH) platform in maintaining or improving the impact of care provided by aged care service providers to older adults living independently. A total of 195 participants were recruited, with 98 participants allocated to the intervention group with sensor systems installed into their homes.

### 2.2. Ethical Approval and Consent to Participate

This study was granted approval by the CSIRO Health and Medical Human Research Ethics Committee (CHMHREC)—Proposal # HREC 4/2018. The participants consented that the information gained during this study could be published and that they would not be identified or have their personal results divulged.

### 2.3. Participants and Recruitment

In total, 195 individuals, aged 65 years and older, were recruited from three aged care service providers in three local government areas (i.e., metropolitan and regional) in Queensland (QLD), Australia. The inclusion criteria were as follows:

- aged 65 years and older;
- living at home—in the care of a designated aged care service provider; and
- English speaking, with proficiency in written English.

The exclusion criteria included the following:

- people residing in long-term residential care;
- Individuals not able to give informed consent due to reasons such as severe cognitive impairment (must have been able to provide informed consent); and
- people who were unwilling to leave their electricity on overnight.

Within each area, simple randomization was performed, and participants were assigned to either a provider’s traditional care (Control, *n* = 97) or had their services designed and delivered through the SSH platform (Smart Home Group) aligned to Consumer Directed Care (Intervention, *n* = 98). All participants assigned to the Smart Home Group received the SSH kit and were able to access the SSH app for up to 12 months.

**Figure 1:**
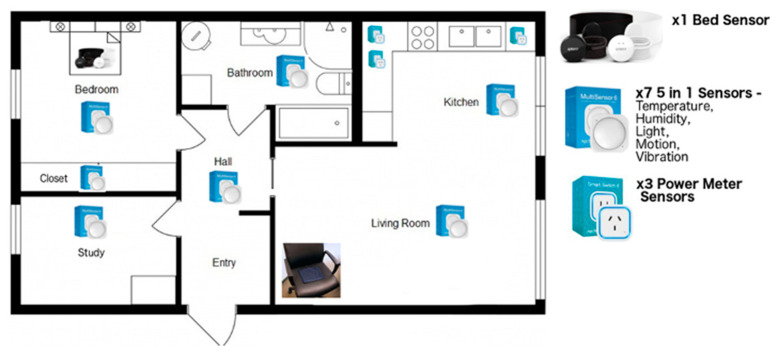
Sensor set-up.

The platform included a sensor-based in-home monitoring system (data collection), a cloud computing server (data analyses), and a client module (data presentation) with a tablet app, a family portal, and a service provider portal. Table 1 demonstrates the sensor types that were used to measure the different types of daily living event. Figure 1 provides a pictorial of where the sensor types were positioned within each participant’s home.

### 2.4. Sleep Monitoring Device

A contact-free sleep monitoring device, EMFIT QS, was used in DACS in order to track the potential changes in an individual’s sleep for up to 12 months.

**Figure 2:**
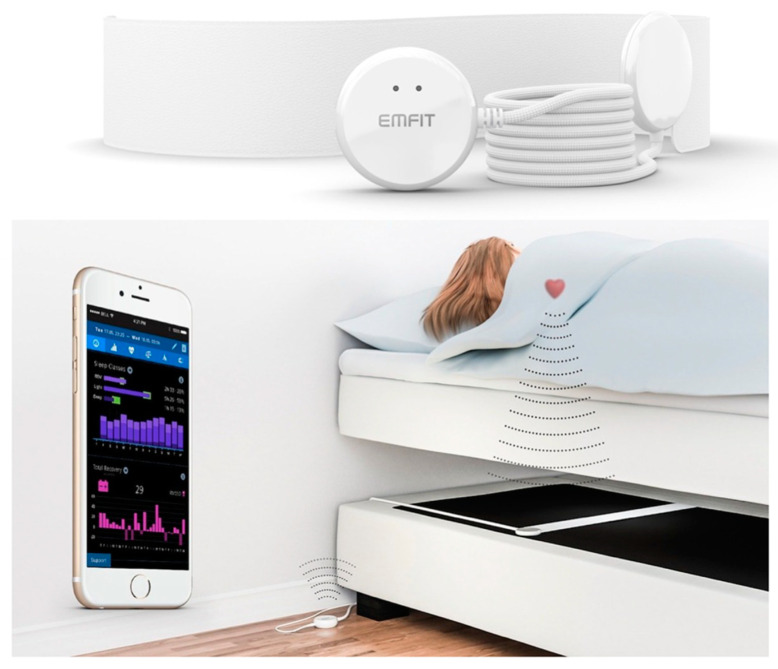
EMFIT QS Sensor (reproduced with permission from EMFIT Ltd.).

The sensor was placed under the bed mattress (Figure 2) [16]. It was combined with a cloud-based analysis platform. The EMFIT QS measures BCG, respiration, and gross body movements and provides overnight activity summary and sleep stage estimation with sleep quality indicators. The HR and RR are calculated in real time with a short dynamic window that automatically discards artefacts. The cloud receives the vital values in 4-second resolution.

### 2.5. Sleep Measurements

The following equivalent measures from sleep summary data of EMFIT QS per night of sleep were derived: total sleep time (TST), measured in hours; sleep onset latency (SOL), measured in minutes; wake after sleep onset (WASO), measured in minutes; sleep efficiency (SE), as a percentage ($\frac{\mathrm{TST}}{\mathrm{Time}\;\mathrm{in}\;\mathrm{Bed}}\times 100$); and time to bed and time out of bed per night of sleep.

### 2.6. Study Population and Period

For the purpose of this pilot study, we only focused on participants from the Smart Home Group, whose sleep was monitored pre-lockdown (November 2019–February 2020) and during (March 2020–May 2020) the COVID-19 hard lockdown in QLD. The lockdown measures for the geographic area from which participants were recruited included no unnecessary visitors inside homes; the allowance for home deliveries to continue, but with packages left at the front door; and the ability to attend shops for essential daily items such as groceries or medical appointments. All non-essential services were ceased, and this included the shutdown of any social type activities. These two periods have been labelled as “pre-lockdown” and “during-lockdown” in our experimental design.

Preliminary assessment and validation of EMFIT QS sleep measurements showed that when someone shared their bed, sleep measurements extracted from the targeted individual can be highly impacted by another person sleeping on the same bed due to the high sensitivity of the EMFIT QS. Therefore, this cohort included only single sleepers, excluding sleepers who shared a bed, in order to avoid any data contamination from co-sleepers.

### 2.7. Statistical Analysis

Statistical analyses were performed in Python, 3.6. Alpha was set at 0.05 and tested two-sided. All continuous variables were assessed for normal distribution by inspection of histograms and the Shapiro–Wilk test. Normally distributed data are shown as mean ± SD. Non-normally distributed data are presented with median and interquartile ranges (IQR). A paired samples *t*-test was performed to compare normally distributed outcome measures between two periods (shown as mean ± SD); non-normally distributed outcome measures were assessed with Wilcoxon signed rank tests.

## 3. Results

### 3.1. Baseline Participant Characteristics

After excluding individuals who shared their bed, 31 participants (54.8% women; average age of 84 ± 6.8 years) were included in the analysis. Population characteristics are summarized in Table 2.

### 3.2. Distribution

**Figure 3:**
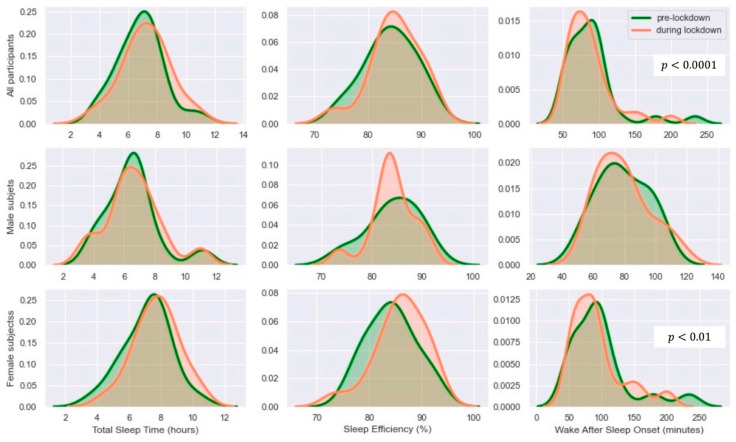
Distribution of total sleep time, sleep efficiency, and wake after sleep onset for all participants, as well as female and male subgroups. Those with indicated *p* violated the normality assumption through Shapiro–Wilk test.

Figure 3 shows that total sleep time and sleep efficiency across the two periods, pre-lockdown and during lockdown, are normally distributed when compared for all participants, as well as female and male subgroups. However, wake after sleep onset for both periods violates the assumption of normality in all participants (*p* < 0.0001) and female groups (*p* < 0.01).

### 3.3. Changes in Sleep Measures before and during Lockdown

**Figure 4:**
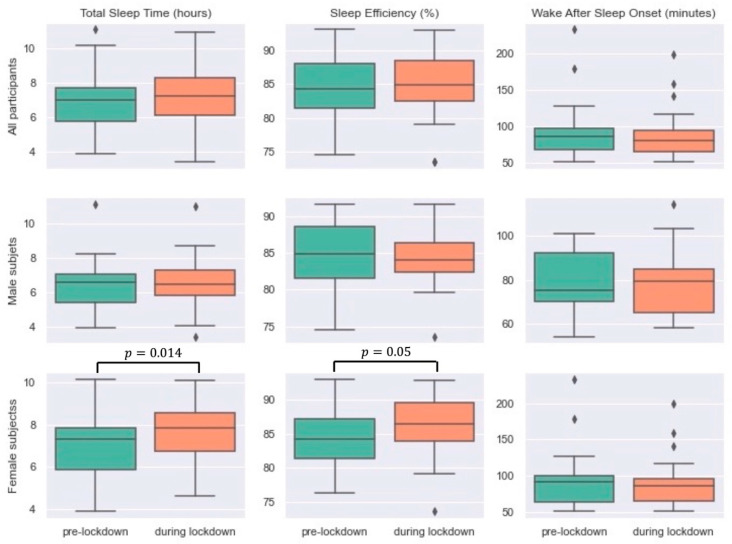
Boxplots of total sleep time (hour), sleep efficiency (%), and wake after sleep onset (min) for all participants, males, and females across two periods of time: pre-lock down and during lockdown.

Comparing the sleep measures of all participants showed that TST was increased by 18 min, SOL remained stable, and there was a slight increase and decrease in SE and WASO, respectively, during the lockdown period. However, none of these changes were significant across the two periods (Table 3). The same pattern was observed when only looking at female participants, with statistically significant differences in TST (a 36-min increase during the lockdown from 6.9 to 7.2 h) and SE (1.7% increase during the lockdown from 84.2% to 85.9%). The male subgroup spent slightly more time awake in bed after sleep onset during the lockdown (from 78.7 to 79.3 min); however, this was not clinically or statistically significant. There was no significant change in sleep onset latency in this cohort before and during the lockdown. Figure 4 shows the box plots of TST, SE, and WASO across the two periods.

There was a non-significant sleep phase advancement in sleep for the cohort overall upon comparing time to bed pre-lockdown and during lockdown—from bedtime 20:11 to 20:25. Time out of bed was similar when comparing the two time periods. For women, however, there was a significant phase advancement, with time to bed moving forward by 36 min during the lockdown, but no significant changes in the time out of bed. This indicates that females spent more time in bed during the lockdown.

## 4. Discussion

Overall, there were no significant changes in measured sleep quantity for this small cohort of older Australians before and during the COVID-19 pandemic lockdown. A non-significant trend towards 18 min of increased total sleep time for the cohort was observed. For older women, a significant increase in total sleep time of 36 min was observed over the study period. This was likely due to an earlier bedtime during the lockdown (March–May 2020) period (average time to bed was 20:47) compared with the pre-lockdown period (November 2019–February 2020) (average time to bed was 20:10). Sleep efficiency remained stable across the pandemic period (between 84%–85%), and there was no significant increase in wake after sleep onset and sleep onset latency. The hypothesized deterioration in sleep quality was not observed in this cohort, which is not reflective of worldwide self-reported changes to sleep quality during the pandemic.

Internationally, sleep and mental health have deteriorated throughout the pandemic. Large international datasets have demonstrated increased time in bed and a delayed sleep phase in pandemic-impacted countries, such as China [17], Italy [7,8], Germany [18], and the United States of America [19]. Despite an increased opportunity for sleep, self-reported sleep quality has deteriorated, with increased reports of poor sleep quality [7]. Casagrande et al. [7] found that 57.1% of 2291 Italian adults self-reported poor sleep quality and increased sleep onset latency compared with pre-pandemic control data. These differences were worse in women compared to men [7]. Over 2020, Google searches for “insomnia” increased 58% over early 2020 compared with the same time period in the previous 3 years [20], and the rate of searches increased as case-fatalities increased worldwide. These studies, however, have tended to consist of younger respondents, with less robust data for older adults. In previous large-scale traumatic events, such as the 2011 earthquake and tsunami, older adults had a higher rate of sleep disturbance, and this was particularly increased in those with financial hardship, poor social support, and pre-morbid anxiety and depression [21].

The Australian response to COVID-19 predominantly consisted of closures and physical distancing measures, called into effect in March 2020 as local case numbers exceeded 1000. However, due to international boarder closures and the success of these early measures, Australian case number have consistently remained with an incident rate of only 29.32 per 100,000 people compared with the UK or South Korea at 6821.82 and 208.5 per 100,000 population, respectively [22]. COVID-19-related mortality in Australia has remained low, at 3.64 deaths per 100,000 population, compared with the UK, at 191.2 deaths per 100,000 population [22]. Thus, the psychological impact of lockdowns and the pandemic in Australia have some unique qualities compared with countries that have experienced a greater impact.

Some gender differences in sleep have emerged in the literature. At the beginning of the pandemic, women were more likely to report poor quality sleep and insomnia [7,23]. However, it has been shown that over a seven-week COVID-19 lockdown, women self-reported improvements in insomnia and sleep quality [23]. This cohort, however, was young, with an average age of 34 years. Men in this cohort were more likely than women to have an exacerbation of insomnia across the lockdown period [23]. The authors postulated gender-based differences in stress response.

In the present study, objective measurements of sleep demonstrated a significant increase in sleep time for older Australian women during the lockdown period. Unlike international data, this cohort achieved increased total sleep time from going to bed earlier. A reduction in care responsibilities for older women may be a possible explanation for the increased total sleep time observed in our cohort. In Australia, before the pandemic, female grandparents have been observed to be more likely to have care responsibilities than male grandparents from both childcare provision and care of another older adult [24]. Among grandparents involved in childcare responsibilities in Australia, grandmothers involved in childcare have been shown to spend 13 h per week compared with grandfathers who spend 10 h per week [24]. During the pandemic, grandparents were generally isolated from their families, including their usual childcare duties. The decrease in caring responsibilities, plus the halt in social activities, is a possible explanation for having more time available for sleep. The other possible explanation for a longer sleep duration during the lockdown (autumn in Australia) could be seasonal fluctuations [25,26].

Objective sleep measurement across the pandemic adds to the large self-report evidence base, as subjective and objective measures do not necessarily align [7]. These data provide some reassurance that sleep efficiency and duration can be maintained, even in a group vulnerable to pandemic-related stress. From a clinician perspective, these data provide some reference points to reassure patients that whilst perceptions of sleep quality change with stress, objectively measured parameters can remain stable. This can assist clinicians in providing insomnia prevention advice, such as avoiding prolonged time in bed when not asleep.

There are several limitations of this pilot project. The sample size was small, and there were no paired subjective measures of sleep quality, such as Insomnia Severity Index and Pittsburgh Sleep Quality Index. Further, the analysis has not included potential confounders including detailed demographics (ethnicity, level of education), medical comorbidities, or level of social support due to small sample size. Additionally, there is little known about the accuracy and sensitivity of measurements captured through the mattress-based devices in older adults. Having said that, longitudinal error was internally controlled, as participants were their own control in this study. Moreover, such devices are easy to set up and use with minimal disruptions to daily life routines (i.e., high practicality), which makes them a preferable option for longitudinal monitoring. This is an extra bonus when used among older populations. The other strengths include objective sleep measurement, which has not been widely performed, and the focus on single sleepers to reduce the risk of erroneous sleep measurement, compared with other studies that have included mobile phone app data, which may have been contaminated by co-sleepers.

Non-wearable and non-invasive monitoring devices, such as EMFIT QS, have advantages for continuous monitoring of older adults’ sleep compared to wearables with limited battery life or mobile phone apps, for which there is a reliance on individuals to trigger or sync a mobile phone app. This study presented a novel application of a home-based sleep monitoring device for a group of people who are vulnerable to chronic diseases. Moving forward, should the pandemic continue to fluctuate and our communities continue to experience long periods of lockdown and isolation, it would be interesting to undertake longitudinal objective sleep measurement with paired subjective sleep measurement in order to better understand the impact of large-scale traumatic events on sleep and daytime function. Furthermore, there is a need for an in-depth validation and acceptability of such mattress-based monitoring devices. Another future direction is to investigate how such novel platforms can provide opportunities for personalized healthcare.

## 5. Conclusions

Older adults have been through a period of increased psychological stress related to COVID-19, which has been associated with a worsening of self-reported sleep quality. This study evaluated objective sleep measures over the pandemic period in a cohort of 31 single sleeping adults aged over 65 years old. There was no significant change in total sleep time; however, women did have a significant increase in total sleep time (36 min), without any deterioration in sleep efficiency. Results of this pilot project provide some reassuring insights into objective sleep measures during an unprecedented time—we can reassure older adults that this is not associated with worsening objective sleep quality.

## Figures and Tables

**Table 1:** Sensor types and settings.

Daily Living Event	Sensor Type	Location
Meal preparation	Motion sensor Electrical power sensor Accelerometers	Dining room Kitchen
Dressing	Motion sensor Accelerometer	Wardrobe
Hygiene	Motion sensor Humidity sensor Temperature sensor	Bathroom
Indoor walking	Motion sensor	All rooms
Sit–stand transition times (out of a bed/chair)	Accelerometer Pressure sensor Sleep sensor	Bedroom Living room

**Table 2:** Participants’ characteristics at baseline.

	Total, *n* = 31
Age, years, mean ± SD [range]	84 ± 6.8 [70–94]
Female, *n* (%)	17 (54.8%)
Body mass index, kg/m^2^, mean ± SD	28.4 ± 6.1

**Table 3:** Changes in sleep measures pre-lockdown and during lockdown.

	Pre-Lockdown			During Lockdown			*p*		
	All	Males	Females	All	Males	Females	All	Males	Females
**TST (h)**	6.9 ± 1.6	6.5 ± 1.8	7.1 ± 1.5	7.2 ± 1.7	6.7 ± 1.9	7.7 ± 1.4	0.06	0.7	0.014 *
**SOL (min)**	25.8 ± 4.9	25.7 ± 4.6	25.8 ± 5.3	25.7 ± 6.6	25.8 ± 6.6	25.6 ± 6.7	0.94	0.9	0.88
**WASO (min)**	89.1 ± 37.3	78.7 ± 16	97.6 ± 47.2	86.8 ± 32.4	79.3 ± 17	93 ± 40.5	0.46	0.82	0.27
**SE (%)**	84.2 ± 4.8	84.2 ± 5.2	84.2 ± 4.7	85.1 ± 4.7	84.1 ± 4.5	85.9 ± 4.9	0.18	0.95	0.05 *
**Time to bed**	20:11:25 ± 06:13:59	19:27:04 ± 07:58:38	20:47:56 ± 04:30:08	20:25:47 ± 05:00:48	20:44:06 ± 05:38:37	20:10:42 ± 04:35:37	0.76	0.47	$<0.1\times 10^{-6}$*
**Time out of bed**	07:01:26 ± 01:32:25	06:54:57 ± 01:33:5	07:06:46 ± 01:33:41	06:51:18 ± 01:22:02	06:41:02 ± 01:30:09	06:59:45 ± 01:16:27	0.19	0.21	0.53

* *p* < 0.05 was considered statistically significant and *p* = 0.05 was considered borderline. TST: total sleep time (hour); SOL: sleep onset latency (min); WASO: wake after sleep onset (min); SE: sleep efficiency (%).

## Data Availability

The data that supports the findings of this study are available upon request.
